# 3D bioprinted mesenchymal stromal cells in skin wound repair

**DOI:** 10.3389/fsurg.2022.988843

**Published:** 2022-10-14

**Authors:** Yuansen Luo, Xuefeng Xu, Zhiming Ye, Qikun Xu, Jin Li, Ning Liu, Yongjun Du

**Affiliations:** Department of the Second Plastic and Aesthetic Surgery, the First People’s Hospital of Foshan, Foshan, China

**Keywords:** skin tissue regeneration, biological engineering, 3D bioprinting, mesenchymal stromal cells, paracrine

## Abstract

Skin tissue regeneration and repair is a complex process involving multiple cell types, and current therapies are limited to promoting skin wound healing. Mesenchymal stromal cells (MSCs) have been proven to enhance skin tissue repair through their multidifferentiation and paracrine effects. However, there are still difficulties, such as the limited proliferative potential and the biological processes that need to be strengthened for MSCs in wound healing. Recently, three-dimensional (3D) bioprinting has been applied as a promising technology for tissue regeneration. 3D-bioprinted MSCs could maintain a better cell ability for proliferation and expression of biological factors to promote skin wound healing. It has been reported that 3D-bioprinted MSCs could enhance skin tissue repair through anti-inflammatory, cell proliferation and migration, angiogenesis, and extracellular matrix remodeling. In this review, we will discuss the progress on the effect of MSCs and 3D bioprinting on the treatment of skin tissue regeneration, as well as the perspective and limitations of current research.

## Introduction

Skin is the first barrier to protect us from invading pathogens and environmental challenges. However, skin tissue injury is common due to trauma and pathological situations such as diabetes mellitus and vascular disorder (1). Wound healing is a complex process which approximately may divide into three phases: inflammation, proliferation, and extracellular matrix (ECM) remodeling (2–4). Multiple cell types, such as platelets, neutrophils, macrophages, fibroblasts (FBs), and myofibroblasts, take part in the skin wound healing process, regulated by biological factors (5). As a result of various reasons, including physiological inflammatory, infection, or systemic diseases, it turns into a chronic wound, which brings an enormous economic burden and influences the population's health (6, 7). Over the decades, the field of skin tissue regeneration has made progress in acute and chronic wound healing. However, there is not enough solid evidence to support general therapeutic modalities that noticeably improve wound healing, including negative pressure or hyperbaric oxygen therapy. Further studies are needed to figure out a superior therapeutic method of skin wound healing (8).

**Table 1:** Research about 3D-bioprinted MSCs in skin tissue regeneration.

Bioprinting strategies	Bioinks and seeded cell	Biological features	References
LIFT	Blood plasma/alginate and FBs/KCs/BMSCs/ADSCs	Bioprinted cells maintain the ability to proliferate and did not show an increase in apoptosis or DNA fragmentation.	(29)
	HA–fibrinogen and ADSCs/ECFCs	Bioprinted cells trigger the development of stable vascular-like networks by direct cell–cell contacts in vascular endothelial growth factor-free medium.	(129)
	Fibrin-collagen gel and AFSs/BMSCs	Bioprinted cells increased wound healing and angiogenesis in full-thickness skin mice wounds due to secreted trophic factors.	(30)
Extrusion and inkjet printing	S-dECM and EPCs/ADSCs	Bioprinted cells accelerated re-epithelization, neovascularization, and wound healing *in vivo*.	(130)
Extrusion-based	Collagen/alginate and ADSCs	Bioprinted cells accelerated wound contraction and healing with faster epithelization and formation of a multilayered epidermis *in vivo*.	(124)
	Gelatine/alginate/PMNT and UC-MSCs	Bioprinted cells accelerated the healing of full-thickness excisional wounds with preferable cell proliferation in the nutritionally deficient environment.	(11)
	Gelatine/alginate/SNAP and ADSCs	Bioprinted cells enhanced the migration and angiogenesis of HUVECs, as well as promoted severe burn wound healing through increased neovascularization *via* the VEGF signaling pathway.	(125)
	Alginate and BMSCs	Bioprinted cells maintained stemness and proangiogenic properties, with more extracellular vesicles.	(123)
	GelMA/curcumin and ADSCs	Bioprinted cells mitigated AGE/AGER/p65 axis-induced ROS and apoptosis, as well as promoted cell survival and diabetic wound healing *in vivo*.	(10)
Core–shell (c/s) extrusion-based	GelMA/succinylated chitosan/dextran aldehyde (D) and BMSCs/HUVECs	Bioprinted cells show cord-like, natural microvascularization and promote skin wound healing *in vivo.*	(126)
	Gelatin/chitosan and MSC (T0523)/HUVECs	Bioprinted cells increased the secretion of wound healing factors EGF, MMP-9, TGF-α, PDGF, decrease pro-inflammatory factor IL-6, and promote neovascularization *in vivo*.	(127)

MSCs, mesenchymal stromal cells; FBs, fibroblasts; KCs, keratinocytes; BMSCs, bone marrow stromal cells; ADSCs, adipose-derived stromal cells; LIFT, laser-induced forward transfer; ECFCs, endothelial colony-forming cells; dECM, decellularized extracellular matrix; EPCs, endothelial progenitor cells; UC-MSC, umbilical cord-derived mesenchymal stromal cells; GelMA, gelatin methacryloyl; HUVEC, human umbilical vein endothelial cells; EGF, endothelial growth factor; TGF-α, transforming growth factor-α; PDGF, platelet-derived growth factor.

Mesenchymal stromal cells (MSCs) make a novel and effective contribution to wound healing. They can be obtained from bone marrow, adipose, umbilical cord tissue (9–11), etc. Unlike embryonic stromal cells (ESCs) or induced pluripotent stromal cells (iPSCs), MSCs are easy to isolate from original tissues with less severe ethical issues and a lower risk of teratoma formation (12, 13). Both clinical and preclinical studies suggested that MSCs can accelerate re-epithelization, neovascularization, and collagen deposition to promote wound healing (14, 15). Recently, MSCs have been introduced as a new treatment for wound healing because of their biological characteristics and paracrine function, which could secrete various bioactive factors, such as vascular endothelial growth factor (VEGF), hepatocyte growth factor (HGF), transforming growth factor β1 (TGF-β1), KGF, IL-8, and IL-6 (16, 17). Moreover, as a cell-free therapy, extracellular vesicles (EVs) derived from MSCs have become a promising wound healing treatment. Extracellular vesicles contain abundant messenger RNA (mRNA), microRNAs (miRNAs), and long noncoding RNA (LncRNA) to regulate the activity of host cells and promote wound healing, avoiding risks related to cell transplant (18). Exosomes derived from MSCs have been confirmed that remarkably promote angiogenesis (19, 20), increase collagen synthesis (21), and expression of growth factors (22) in wound healing. Despite all the advantages of MSCs therapy, there are still difficulties, such as the limited proliferative potential and the biological processes that need to be strengthened.

Recently, three-dimensional (3D) bioprinting as additive manufacturing technology has been applied to fabricate tissues/organs to achieve the controllable spatial distribution of living cells and biological materials (23–25). With biocompatibility biomaterials, such as decellularized extracellular matrix (dECM), alginate and hyaluronic acid, 3D-bioprinted cells have been used for tissue repair (26). In the field of skin tissue regeneration, 3D-bioprinted bioengineered skin grafts containing FBs, endothelial cells (ECs), or human dermal fibroblasts (HDFs), have been proved could enhance skin wound repair (27, 28). In recent years, a variety of research explored the potential of MSCs as effective seed cells for the treatment of skin wound healing. 3D-bioprinted MSCs maintained a better ability to proliferate increased biological factors to promote skin wound healing (29, 30). With their ability to differentiate and paracrine effect, MSCs show infinite potentialities based on 3D bioprinting technology (31).

Accordingly, in this review, we will discuss current knowledge about the role of MSCs and 3D bioprinting in the treatment of skin wound healing, as well as the perspective and limitations of recent research.

## The process of skin wound repair

Following injury, mammalian wound healing is traditionally divided into three phases: inflammation, proliferation, and ECM remodeling (32). The first stage of wound healing, inflammation, will happen from the moment of tissue damage. Platelets, neutrophils, macrophages, and fibrin matrices work together to prevent ongoing blood and fluid losses as well as infection. In contrast, macrophages are considered vital for coordinating other issues in the wound healing process (33). The second stage, proliferation, is attributed to the proliferation and migration of fibroblasts, myofibroblasts, and keratinocytes. These different cell types are associated with angiogenesis and re-epithelialization to restore the barrier function of the epidermis (2, 34). In the third stage, ECM remodeling, all the activities after injury cease. Macrophages and myofibroblasts will undergo apoptosis, leaving collagen and other extracellular matrix proteins (35). Matrix metalloproteinases secreted by macrophages, fibroblasts, and endothelial cells strengthen the repaired tissue (36). However, the incongruity of any stage will occur in chronic wounds or keloid scar formation (37).

Chronic wounds or impaired wound healing are defects in the skin for more than 6 weeks (38). Typically, chronic wounds may be divided into three types: vascular dysfunction, diabetes, and pressure ulcers (39). Inappropriate physiological inflammatory reactions, underlying systemic diseases, such as diabetes mellitus and vascular disorders, and infections will lead to impairments of cell proliferation and migration and extracellular matrix damage (6, 8). The formation of myofibroblasts is compromised, partly due to hypoxic conditions or vascular insufficiency, which will contribute to the lack of granulation tissue and delayed wound healing (40).

Unfortunately, damaged tissue could not regain the properties of unwounded tissue (41). Importantly, nonhealing and dysfunctional healing cause lifelong disability and a significant economic burden (42). Therefore, the emphasis of research should be on enhancing wound healing and regeneration of original tissues in the future.

## MSCs and skin wound healing

**Figure 1:**
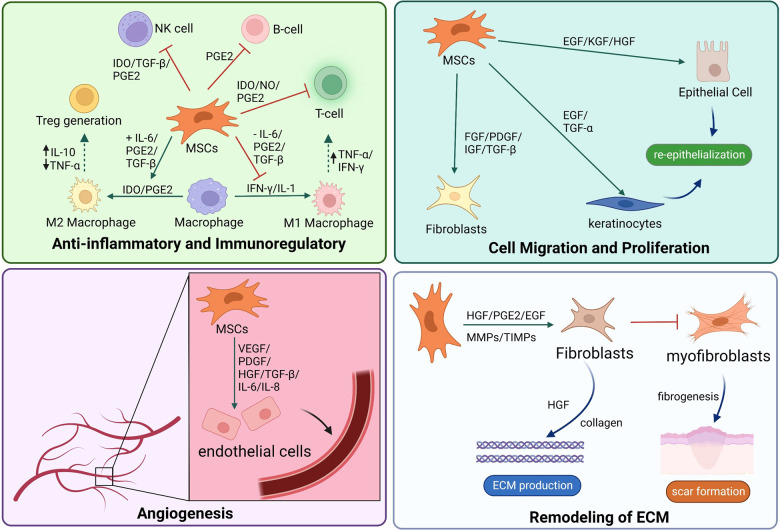
MSCs promote skin tissue regeneration through anti-inflammatory and immunoregulatory, cell migration and proliferation, angiogenesis, and ECM remodeling. MSCs, mesenchymal stromal cells; ECM, extracellular matrix.

Recently, MSCs have become a promising therapy in the field of regenerative medicine because of their pluripotency, self-renewal, and paracrine of biological factors (43). MSCs, possibly derived from the mesoderm, could differentiate into various mesenchymal tissue lineages, such as chondrocytes, adipocytes, osteoblasts, and even myoblasts (44). The most MSCs used in skin regeneration are adult stromal cells because of less ethical controversy and substantial legal restrictions (45). MSCs would accelerate skin wound healing by regulating multiple phases of wound reconstruction, including inflammatory response, cell proliferation, wound angiogenesis, and wound remodeling (46). Fifty years ago, Friedenstein et al. have been isolated bone marrow stromal cells (BMSCs) from bone marrow (47). In the last few decades, adipose-derived stromal cells (ADSCs) have been a new source of MSCs introduced to wound healing because they are obtained from adipose tissues with less invasive methods and less ethical concerns (16). Including human umbilical cord-derived mesenchymal stromal cells (HUC-MSCs), MSCs play an important role in wound healing (Figure 1).

### MSCs and anti-inflammatory/immunoregulatory

There is an amount of literature about skin wound healing, pointing out that skin wound healing is a complex process that depends on many cell types. Molecular and cellular mechanisms are critical for the process of cutaneous wound healing. At the early stage of wound healing, keratinocytes and inflammatory cells seem necessary. First, leukocytes, especially neutrophil granulocytes, transmigrate to the injury site to initiate and perpetuate inflammation (48, 49). Inflammation is a self-defense mechanism against noxious stimuli in the early stages of wound healing, with a significant objective of removing necrotic debris and pathogenic microorganisms from the wound bed and controlling local area damage (50). MSCs can cooperate with various immune cells to modulate inflammatory responses such as B cells, T cells, natural killer (NK) cells, neutrophils, and macrophages (51, 52). Moreover, MSCs promote the polarization of macrophages to an M2-like phenotype, which reduces inflammation and immunosuppressive function through a prostaglandin E2-dependent mechanism (53). Recent studies have demonstrated that MSCs encourage the polarization of macrophages toward an anti-inflammatory, reparative M2 phenotype by a paracrine mechanism. It has been reported that MSCs could secrete transforming growth factor beta (TGF-β) (54), C-X-C motif chemokine ligand 12 (CXCL12) (55), tumor necrosis factor-α-induced gene/protein-6 (TSG-6) (56), and prostaglandin E2 (53) to induce macrophage M2 polarization (53, 57). The cell–cell interaction between MSCs and macrophages in the progress of skin wound healing can accelerate skin tissue regeneration (58).

Meanwhile, MSCs also inhibit the proliferation of activated helper T (Th) cells (59). Mo et al. found that MSCs suppressed Th2 inflammation by regulating macrophage activation *via* soluble mediators rather than direct cell-to-cell contact (60). Besides, the extracts of MSCs could suppress Th2 cells and reduce the expression of IL-17 and IFN-γ, which further demonstrate that MSCs inhibit Th2 cells through paracrine factors (61, 62). In clinical, the immunomodulatory ability of MSCs could reverse the ratio of Th1 cells to Th2 cells, with an increase in Th1 and a decrease in Th2 achieving a new balance (63). This interaction could decrease the production of interferon γ (IFN-γ) and interleukin (IL)-17 and increase the production of IL-4 secreted by Th cells, thus leading to T cells polarizing from a pro-inflammatory to an anti-inflammatory phenotype (64).

MSC sheet technology enables cultured MSC harvest without enzymatic treatment or cell or protein disruption using temperature-responsive cell culture dishes (65). With this application, changes in culture temperature cause oscillation between hydrophilic and hydrophobic states. Cells adhere to and proliferate on the surface of the culture dish at 37 °C, and a monolayer cell sheet with ECM detach spontaneously at temperatures below 32 °C without enzymatic digestion (66). Application of MSCs sheet also brings preferable wound healing and less scar formation, believed that it can suppress macrophage infiltration and chemotactic response of macrophages (67, 68). In clinical research, MSCs could reduce the expression of inflammation and oxidative stress-related proteins to improve diabetic foot ulcers (DFU) healing by Nrf2 (69). It has been reported that MSCs therapy may reduce inflammation and has been applied in acute and chronic liver injury, which suggests MSCs improve tissue regeneration using their anti-inflammatory properties (70).

### MSCs and proliferation/migration

The intermediate stage of wound healing contains proliferation and migration of keratinocytes, the proliferation of fibroblasts, matrix deposition, and angiogenesis. Stationary keratinocytes are converted to flat migratory keratinocytes to start re-epithelialization (27). In this stage, fibroblasts stimulate wound healing by proliferating and synthesizing a large amount of ECM components such as collagen and elastic fibers under the stimulation of trauma (71). MSCs stimulate the migration and proliferation of keratinocytes by the expression of epidermal growth factor (EGF) and transforming growth factor-α (TGF-α) (72). The treatment of MSCs improves the survival rate of fibroblasts and enhances the healing effects (73). Evidence shows that BMSCs improve the proliferation and migration of dermal fibroblasts (74). *In vivo*, MSCs increased the expression of CK19 and proliferating cell nuclear antigen (PCNA) and promoted the regeneration of dermal tissue (75, 76). Specifically, PCNA participates in cell proliferation by mediating DNA polymerase while blocking the PCNA production in cells severely affects cell division, which takes a significant part in the synthesis of DNA and its repair, cell proliferation, and progression of the cell cycle (77, 78). Studies show treatment of MSCs could elevate the expression of PCNA, promote wound healing, and enhance re-epithelialization (79–81).

Animals treated with MSCs show improving wound healing with no detectable side effects related to increasing viability, proliferation, and migration of epithelial cells (82). *In vivo*, the application of MSCs could promote re-epithelialization by enhancing the proliferation of epidermal keratinocytes (83). Moreover, MSCs strengthened the dermal and epidermal cell proliferation ability in a dose-dependent manner and positively impacted oxidative stress injury, which could improve cutaneous wound healing (84). All the evidence indicates that MSCs have a positive effect on wound healing.

### MSCs and angiogenesis

Angiogenesis plays an essential role in the process of wound healing. Creating new capillaries will bring oxygen and nutrients to growing tissues and remove catabolic wastes. Therefore, angiogenesis contributes to the repair of wound tissue (85). Angiogenesis is strictly regulated by a variety of factors, mainly through the secretion of proangiogenic factors, such as VEGF and platelet-derived growth factor (PDGF), leading to stimulating endothelial cell proliferation and migration and angiogenesis (86). Rehman et al. found that MSCs secreted synergistic proangiogenic growth factors, such as VEGF and HGF, which enhance angiogenesis (87). The previous review reported that MSCs could promote re-epithelialization, angiogenesis, collagen synthesis, and neovascularization by secretion of multiple growth factors including VEGF, HGF, TGF-β1, KGF, IL-8, and IL-6 during the progress of wound healing (88). MSCs improved cell viability, migration, and angiogenesis of the high glucose-damaged human umbilical vein endothelial cells (HUVECs) through paracrine, increasing the expression of IL-6, TNF-α, ICAM-1, VCAM-1, BAX, P16, P53, and ET-1(89). Different experimental models mimic the effect of MSCs in wound healing, performed preferentially in rodents. Subcutaneously injecting MSCs into the full-thickness wounds of mice will result in more angiogenesis and promote wound healing (90). With treatment of MSCs, the density of neovascularization in wound bed was increased, also with the expression of VEGF, while the expression of IL-10, IL-6, IL-1β, and TNF-α were significantly decreased (91). Meanwhile, MSCs could enhance angiogenesis and improve the survival rate of graft skin *in vivo* (92). MSCs also positively impacted vascular regeneration and endothelial leukocyte adhesion modulation in critical ischemic skin (93).

### MSCs and ECM remodeling

Late-stage healing involves remodeling of ECM, resulting in scar formation and barrier restoration (28). During the wound remodeling stage, fibroblasts differentiate into myofibroblasts, and the granulation tissue gradually becomes fibrotic; collagen gradually increases; the wound begins to contract, and eventually, scar tissue is formed (94). MSCs would release plenty of cytokines and growth factors with anti-fibrotic properties (95, 96). In the early progress, MSCs improve collagen remodeling through synthesizing collagen types I and III of wound healing while reducing scarring in the late stage by inhibiting collagen formation (97). Treated with MSCs, fibroblasts secret more HGF and increase collagen production. With the inhibition of excessive fibrogenesis, fibroblast proliferation, and α-smooth muscle actin expression, MSCs can reduce scar formation during wound healing (98). MSCs also inhibited fibrosis by decreasing the expression of profibrotic genes and protein, promoting extracellular matrix regeneration, inhibiting fibroblast contraction, and reversing myofibroblast activation (99). It has been reported that MSCs were capable of myofibroblast suppression and anti-scar formation *in vivo* and *in vitro* (100). MSCs have been considered significant in skin wound repair. However, further research is still needed to increase the biological regulation of wound healing.

### MSC-EVs and skin wound healing

Although mesenchymal stem cells could regulate inflammation, promote cell proliferation and migration, and improve angiogenesis during skin wound healing, a few disadvantages limit its wide application. Mesenchymal stem cell therapy may have a problem with storage and transportation, as well as the risk of cancer and deformities. As a result, it is not a definitive treatment without a long-term study on safety (101). EVs, including exosomes, have taken part in various pathological physiology processes and significantly contributed to MSCs (102). It has been reported that EVs derived from MSC carried a large number of regulatory factors, such as active protein, miRNA, and lncRNA (103). EVs play a key role in several biological processes that activate downstream target cells through a paracrine effect (104). Like MSCs, researchers have discovered that EVs derived from MSCs could modulate the inflammatory response, accelerate cell proliferation, promote angiogenesis, and regulate ECM remodeling during wound healing (105).

In the early stage, exosome derived from MSCs (MSCs-exo) helps monocytes translate into M1 macrophages through many immunomodulatory proteins released, such as tumor necrosis factor-alpha (TNF-α), macrophage colony-stimulating factor (MCSF) and retinol-binding protein 4(RBP-4) (106). The expression level of oxidative stress-related proteins and inflammatory cytokines is reduced (69). MSC-EVs upregulate the expression of monocyte chemoattractant protein-1and macrophage inflammatory protein-1α reduces early inflammation and oxidative stress (69, 107). In the proliferation stage, MSC-EVs are internalized by fibroblasts as well as epidermal keratinocytes and promote cell migration and proliferation by expression of N-cadherin, cyclin 1, proliferating cell nuclear antigen, collagen type I and III (22). Besides, MSC-EVs are enriched in vascular endothelial growth factor A (VEGF-A), platelet-derived growth factor BB (PDGF-BB), and noncoding RNAs, which promote proliferation and angiogenesis of vascular endothelial cells (19, 108). In the late stage, MSC-EVs prevented fibroblast-to-myofibroblast differentiation by increasing the ratio of collagen III to collagen I and the ratio of TGF-β3 to TGF-β1 to reduce scar formation (109). Therefore, MSC-EVs have become a hot topic in the field of skin wound regeneration as a cell-free therapy.

## Three-dimensional (3D) bioprinting

**Figure 2:**
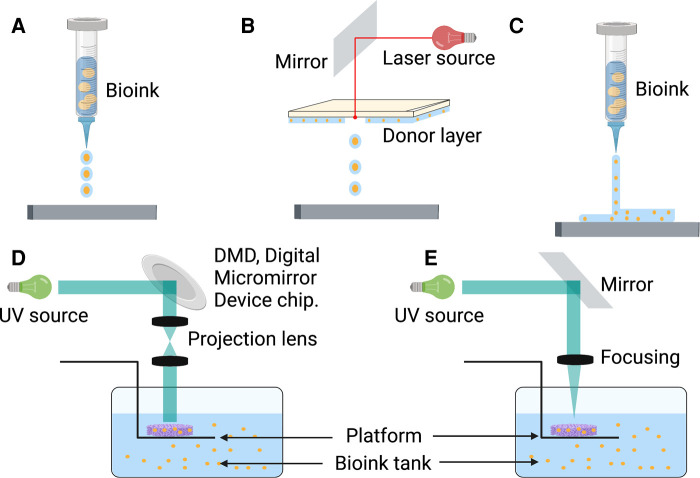
There are multiple strategies of 3D bioprinting, including inkjet bioprinting (**A**), laser bioprinting (**B**), extrusion-based bioprinting (**C**), SLA-based bioprinting (**D**), and DLP-based bioprinting (**E**). DLP, digital light processing; SLA, stereolithography.

Inspired by traditional inkjet printer technology, Thomas Boland directly printed viable mammalian cells onto hydrogel-bases papers with a cell printer, successfully exploring the field of 3D bioprinting technology used in tissue engineering (110). Bioink is a biomaterial composed of living cells, biological factors, and biological glue (111). With sufficient mechanical properties and biocompatibility, bioink can provide a stable and harmless environment of proliferation and differentiation for cells (112). Depending on the type of cell deposition, 3D bioprinting technology can be classified into three main strategies: drop-based, filament-based, and plane-based (113) (Figure 2).

### Drop-based bioprinting

Inkjet or laser bioprinting are two commonly used strategies of drop-based 3D bioprinting. In inkjet-based bioprinting, inkjet bioprinters utilize heat or mechanical compression to create and eject drops. In this bioprinting process, various volumes of ink drops are created based on computer control, in which each drop of bioink contains 10^4^–30^4^ cells (114). Laser-based bioprinting utilizes the laser-induced forward transfer (LIFT) effect to print different biomaterials and living cells (115). During the process, the incident laser light causes the ejection of bioink droplets, which are subsequently received on a receiving substrate (116, 117). It has been reported that inkjet printing can be combined with gene delivery to effectively control stromal cell differentiation while bioprinting neural stromal cells (NSCs) (118). Sorkio et al. used laser-based bioprinting to demonstrate tissues mimicking the structure of the corneal tissue with stromal cells (119). A separate study indicated that MSCs could keep the predefined structure and maintain cell competency for tissue repair (120). Inkjet and laser bioprinting allow the printing of cells, materials, and protein molecules rapidly and inexpensively. However, the smooth printing process would frequently disrupt by the clogging of nozzles because of bioink gelation and the unequal-sized drops in inkjet bioprinting (121). Meanwhile, long-fabrication times and gravitational settling of cells in solution are other challenges in drop-based bioprinting (122).

### Filament-based bioprinting

Extrusion-based bioprinting is the most popular approach in the research of filament-based bioprinting strategy. Based on mechanical driven force (displacement driven) or pneumatic driven force (pressure driven), extrusion bioprinting mechanisms can directly dispense the higher viscosity bioinks out of the biomaterial cartridge (123). During the development of extrusion-based bioprinting, the scaffold of acellular polymers, such as polycaprolactone (PCL), polylactic acid (PLA), and poly lactic-co-glycolic acid (PLGA), was used for 3D cell culture by extrusion printing (124–126). With the different applications of bioinert hydrogel materials such as sodium alginate, extrusion bioprinting can print bioinks of living cells to a particular flow out as seamless circular cylindrical filaments with computer manufacturing (127, 128). Extrusion-based bioprinting device creates a scaffold of adipose-derived mesenchymal stromal cells (ADSCs) in alginate-gelatin (Alg-Gel) hydrogel for tissue repair and regeneration (129). Moreover, this technology can provide a kidney organoid with highly reproducible cell numbers and viability (130). Extrusion-based bioprinting brings a promising future of producing rapid and high-throughput organoids for drug screening, disease modeling, and tissue repair. However, rapid speed and high pressure can enhance shear stress, which decreases cell viability (131). The current research focuses on extrusion bioprinting study intensively on cell-instructive hydrogels as bioinks to provide a cell-friendly microenvironment (132).

### Plane-based bioprinting

Compared with other 3D bioprinting strategies, plane-based bioprinting technology, such as digital light processing (DLP) and stereolithography (SLA) bioprinting, has significant advantages in efficiency, printing resolution, and working conditions (133). DLP-based bioprinting employs projection light to biomaterials to obtain the predesigned structures. In contrast, SLA-based bioprinting achieves photo polymerization by a light pencil scanning the surface of liquid bioink (133, 134). With light-based techniques, DLP/SLA-based bioprinting can print the entire layer with higher accuracy and speed (135). Through a DLP-based 3D printer, Ma et al. printed a 3D triculture hepatic model encapsulated kind of cells, including induced iPSCs and ADSCs. The microstructure can promote maturation and maintain the functions of cells (136). With SLA-based bioprinting technology, the 3D cell-laden hydrogel scaffolds represent high cell viability and cell adhesion (137). Although this technique has shown the characteristics of high precision, fast speed, and mild condition, its running cost, and the lack of compatible bioinks limit its broader applicability (138).

## 3D-bioprinted MSCs in skin tissue regeneration

Traditional 2D cell cultures cannot recreate the native three-dimensional (3D) cell microenvironment, which provides cell–matrix and cell–cell interactions that readjust cell morphology and gene expression (139–141). 3D bioprinting is a promising biofabrication strategy, using living cells and biomaterials as bioink to create artificial multicellular tissues (142). In the field of skin tissue repair, 3D bioprinting is currently being explored in developing more complex synthetic skin models (143, 144). Nowadays, various biomaterials have been widely investigated as scaffolds for bioprinting in tissue engineering and skin wound healing (145, 146). Usually, hydrogel containing FBs, keratinocytes (KCs), and HUVECs are bioprinted as scaffolds directly applied on the wound bed (147). Jin et al. *had* taken advantage of the acellular dermal matrix (ADM) and HUVECs as bioink to 3D-bioprinted functional skin model, which maintained the ECM components to promote cell viability and form the vascular network and framework (148). Recently, 3D bioprinting has been successfully performed using multiple mesenchymal stromal cell types for tissue repair, including cardiovascular, hepatic, and skin (149). 3D-bioprinted MSCs preserved proangiogenic properties and secreted more EVs containing a greater variety of proteins (150). However, not much research focuses on adult stromal cells as seeded cells in skin wound regeneration (Table 1).

In 2010, Koch and colleagues utilized skin cell lines (fibroblasts/keratinocytes) and MSCs as bioink for skin regeneration based on laser printing. MSCs maintained the ability to proliferate and did not show an increase in apoptosis after 3D bioprinting (29). 3D-bioprinted MSCs increased angiogenesis and wound closure rates due to secretion of biological factors rather than direct cell–cell interactions, such as basic fibroblast growth factor (bFGF), and fibroblast growth factor (FGF) and growth differentiation factor (GDF) (30). Moreover, Roshangar et al. evaluated a 3D bioprinting scaffold loaded with MSCs on rat skin burn defects. Data showed that the scaffold promoted wound healing by creating a continuous epidermal layer without scar formation (151).

3D-bioprinted MSCs could improve wound healing *in vivo* by generating collagen and enhancing cell proliferation. Besides, 3D-bioprinted MSCs would maintain a preferable cell proliferation in the nutritionally deficient environment (11). In addition to utilizing hydrogel and MSCs for 3D bioprinting, various bioactive substances have been applied to enhance the abilities of MSCs, such as angiogenesis. A 3D-bioprinted scaffold loaded with MSCs and SNAP, which could release NO, can improve the migration and angiogenesis of HUVECs. On the other hand, the hydrogel scaffolds accelerated the serve burn wound healing by promoting epithelialization and collagen deposition (152). It has been reported that 3D-bioprinted MSCs could accelerate diabetic wound healing by combining bioink with curcumin and gelatin methacryloyl (GelMA). 3D-bioprinted MSCs with curcumin could better exert antioxidant and anti-apoptotic activity to promote wound healing (10). As widely used biological material with biocompatibility and multichannel printing technology, Turner et al. established a regenerative, dual cell delivery 3D core/shell (c/s) “living dressing” system using MSCs. It indicated that the construct provided an appropriate microenvironment to improve the proliferation and differentiation of MSCs (153). In addition, Turner et al. discovered the 3D core/shell MSCs dressing would accelerate angiogenesis and anti-inflammatory to promote wound healing in thermal injury by increasing the expression of wound healing factors and neovascularization EGF, PDGF, MMP-9, TGF-α and decreasing pro-inflammatory factor IL-6 (154).

In the last few decades, new materials and technologies have been developed to fabricate skin substitutes (31). Compared to conventional tissue engineering technologies, 3D bioprinting can deposit different cell types and specific biomaterials with a high spatial resolution (155). In 2011, Gruene et al. utilized endothelial colony-forming cells (ECFCs) and ADSCs to build a layer-by-layer scaffold using laser-assisted bioprinting and discovered direct cell–cell contacts, which may promote angiogenesis (156). Skin tissue contains a variety of cell types. Baltazar et al. have created artificial dermis using bioink containing human foreskin dermal FBs, human ECs derived from cord blood human endothelial colony-forming cells (HECFCs), and human placental pericytes (PCs) suspended in rat tail type I collagen and printed epidermis with human foreskin KCs. The result showed the 3D bioprinting of artificial dermis enhanced the formation of microvessels and the epidermal rete *in vivo* (28). Another research used endothelial progenitor cells (EPCs) and MSCs to 3D bioprint a full-thickness skin model. This model accelerated re-epithelization, wound closure, and neovascularization (157). For skin appendages, 3D-bioprinted MSCs have been confirmed could enhance stemness maintenance by sweat gland (SG) lineage *in vitro* (158). With 3D bioprinting, alginate-gelatin and epidermal progenitors could enhance sweat gland regeneration (159). Through bioprinting an SG-like matrix, MSCs could differentiate into functional SGs and facilitate SGs recovery in mice (160). Despite MSCs and 3D bioprinting technology showing great potential for preparing artificial skin, future research should concentrate on skin tissue bioprinting and make it more adaptable to clinical needs.

## Discussion

The objective of skin tissue regeneration is to realize structural and functional reconstruction, at the same time promoting wound healing and reducing scar formation (161). MSCs have been considered to have a promising potential in skin tissue regeneration with their differentiation abilities and paracrine effect (15). Many studies have shown that MSCs could regulate inflammation, promote cell proliferation and migration, and improve angiogenesis during skin wound healing (15). However, MSCs therapy still has obstacles, such as its low frequency in tissues and the limited proliferative potential (162). Recently, studies have focused on the therapeutic potential of EVs derived from MSCs in skin wound regeneration. MSC-EVs and exosomes are considered to affect skin wound healing significantly. Unlike MSC-based therapy, MSC-EVs therapy has advantages in delivery and storage, as well as a lack of endogenous tumor-formation potential (163). One of the most significant advantages of MSC-EVs therapy is the possibility to inject EVs locally, thus minimizing the side effects of cell administration (102). Nevertheless, clinical applications of MSC-EVs require a long-term study on safety to prevent the development of uncontrolled immunosuppression in MSC-EVs recipients (164). Although evidence suggests the therapeutic potential of MSCs and MSC-EVs, there are still further studies to be done before MSCs and MSC-EVs could be offered as a common clinical therapy for skin wound healing.

On the other hand, with the application of 3D bioprinting, MSCs have more ability to proliferate and secrete biological factors to enhance cell–cell interaction. As 3D bioprinting technology developed, *in situ* bioprinting or “*in vivo*” bioprinting has been applied to tissue regeneration, including skin, cartilage, and bone (165). It's reported that autologous dermal fibroblasts and epidermal keratinocytes, along with the fibrinogen-collagen hydrogel, were directly printed into the wound of a porcine model, with better re-epithelialization and reducing the healing time (147). Besides, through cellular self-assembly bioprinting, upregulated expression of tissue-specific functional genes indicated increased tissue functionality to realize multitissue organs-on-a-chip with different cell types (166). There is substantial evidence supporting various methods of MSCs culture influence the release of EVs, while little research concerned EVs derived from 3D-bioprinted MSCs (167). According to a recent study, Chen et al. combined 3D core–shell bioprinting and MSCs to increase MSC-derived EVs’ production (150). In his platform, the 3D-bioprinted MSCs enriched particles by ∼1,009-fold compared to traditional 2D culture, expressing higher stemness markers and preserving proangiogenic properties. Moreover, 3D-EVs contained hundreds of unique protein profiles compared to 2D-EVs. Supported by 3D bioprinting technology, the bioprinted 3D structures loaded with EVs recapitulated the blood-perfused microvessels with a new functional vasculature *in situ* (168). Besides, Bari had found that 3D-bioprinted MSC-EVs could release the secretome from the scaffold with a fast speed for tissue regeneration (169). Moreover, the release was governed by the scaffold shape and the application of different biomaterials, for example, increasing alginate concentration or cross-linking with protamine. Technological developments may present new opportunities and challenges for MSCs in skin tissue regeneration.

Although MSCs and 3D bioprinting technology bring a new therapeutic strategy for skin wound healing, some obstacles still need to be overcome. The skin is one of the most vital organs as a protective barrier against various external agents (170). At present, the mechanical strength of 3D bioprinting hydrogels is insufficient, which could not be satisfied with the unique physical and mechanical characteristics of human skin. In addition, the skin contains various appendages, such as sweat glands, adipose glands, hair follicles, and blood vessels, along with nerve endings (171). Despite the sweat gland regeneration function of 3D bioprinting MSCs, 3D bioprinting skin tissue could not simulate every structure or functional reconstruction, especially nerve regeneration.

**Figure 3:**
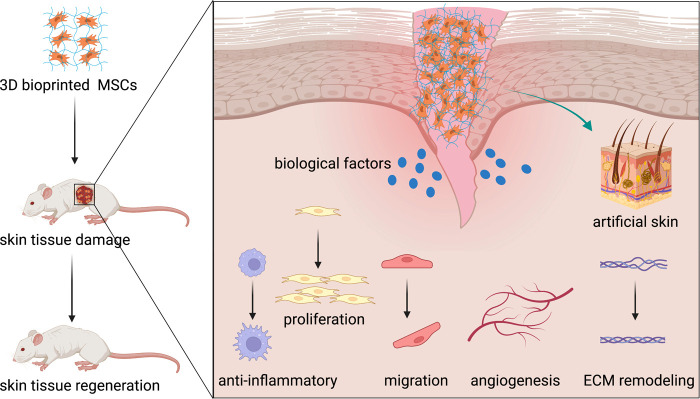
3D-bioprinted MSCs promote skin tissue regeneration. MSCs, mesenchymal stromal cells

After all, 3D-bioprinted MSCs have played a positive role in skin wound healing. With the progress of related technologies and the application of new biomaterials, 3D bioprinting will hopefully overcome the difficulties mentioned above and make a big difference in skin tissue regeneration (Figure 3).

## Conclusion

Wound healing and skin tissue regeneration have been critical clinical issues for decades. Various methods have been used to promote skin wound healing by better regulating every phase of wound healing. As depicted in the published studies, 3D-bioprinted MSCs are a promising therapeutic strategy for skin tissue regeneration because of their preferable differentiation and paracrine effect of biological factors. However, interdisciplinary collaboration is still needed to overcome the difficulties, such as the mechanical strength and skin appendages of bioprinted MSCs. In conclusion, 3D-bioprinted MSCs have been proven to have a positive role in skin tissue regeneration. Further studies are needed to assess the long-term outcomes and well-designed clinical studies to apply this strategy in clinical medicine.
